# Assessment of quality of life among professional palliative care providers in Tabuk City, Saudi Arabia: A cross-sectional study

**DOI:** 10.1097/MD.0000000000051149

**Published:** 2026-10-23

**Authors:** Hanan Mohammad H. AlJammaz, Badaya Awadh M. Alshehri, Maram Ati Almohammadi, Asma Ali Alharbi, Fahad Abdullah Alqahtani, Ahmed Raja Albalawi, Rasha Ali Abdelrahman, Ahmed Abrahem Alhweti

**Affiliations:** a Family Medicine Department, Family Medicine Resident, Tabuk Health Cluster, Tabuk, Saudi Arabia; b Family Medicine Department, Family Medicine Consultant, Tabuk Health Cluster, Tabuk, Saudi Arabia; c Saudi Fellowship in Hematology, Oncology Department, King Fahad Specialist Hospital – Tabuk, Tabuk, Saudi Arabia; d Saudi Fellowship in Diabetes Management, Diabetic Center, Consultant of Family Medicine and Diabetologist, Tabuk Health Cluster, Tabuk, Saudi Arabia; e Family Medicine Department, Trainer and Quality Coordinator, Family Medicine Residency Training Program, Tabuk Health Cluster, Tabuk, Saudi Arabia; f Family Medicine Department, Family Medicine Senior Registrar, King Salman Armed Forces Hospital, Tabuk, Saudi Arabia.

**Keywords:** health professional, palliative care, quality of life, Saudi Arabia, WHOQOL-BREF

## Abstract

This study aimed to assess the quality of life (QOL) among palliative care professionals in the Tabuk region of Saudi Arabia and to identify the factors associated with it. This cross-sectional study was conducted between January and September 2025 among 277 palliative care professionals of both sexes and different specialties at Tabuk Health Cluster Hospitals and King Salman North West Armed Forces Hospital, Tabuk City, Saudi Arabia. The English version of the World Health Organization QOL assessment-BREF (WHOQOL-BREF) was employed for data collection. The questionnaire was converted to an online survey through the Google Forms platform. The survey link was disseminated to the study population via social media applications. The study outcomes included physical health, psychological health, social relationships, and environment domains. After calculation of QOL transformed scores, they were compared across participants’ characteristics by independent samples *t* test or 1-way analysis of variance test, as appropriate. The mean age of participants was 33.8 ± 6.3 years. More than half (56.7%) held a bachelor’s degree, and nurses represented the largest group (48.4%). By specialty, internal medicine was most common (35.4%), followed by critical care (21.3%). Among QOL domains, physical health had the highest mean score (51.7 ± 14.0), while the social domain had the lowest (45.9 ± 21.8). Psychological (48.7 ± 17.0) and environmental (48.6 ± 16.4) domains showed intermediate scores. Job type was significantly associated with physical health (*P* = .037), with patient care technicians scoring lower (45.0 ± 13.9) than specialists (55.1 ± 12.6). Specialty differences were significant for social (*P* = .027) and environmental (*P* = .005) domains: palliative medicine scored highest in social (53.8 ± 24.7) and environmental (56.6 ± 19.0), while anesthesia scored lowest in both (36.9 ± 19.9 and 41.6 ± 20.0, respectively). Palliative care professionals reported an overall moderate QOL, with the lowest scores observed in the social domain. This finding may reflect the emotional demands and workload associated with palliative care practice. The observed differences across job type, specialty, health status, marital status, and healthcare sector suggest that QOL may be associated with both professional and personal factors, with certain subgroups reporting lower QOL and potentially greater vulnerability to occupational and psychosocial stressors.

## 1. Introduction

Palliative care constitutes an integral part of modern healthcare. The International Association for Hospice and Palliative Care (IAHPC) defines palliative care as “the active holistic care of individuals across all ages with serious health-related suffering due to severe illness, and especially of those near the end of life.”^[1,2]^ It aims to improve the quality of life (QOL) of patients, their families, and their caregivers. It can be delivered alongside curative treatment and commonly targets symptoms such as pain, anxiety, depression, and fatigue. The demand for such services has grown steadily due to global population aging and the rising burden of chronic diseases.^[3,4]^

Specialized palliative care professionals fulfill 5 primary roles: care provider, care consultant, educator, researcher, and advocate,^[5]^ roles that apply to both nurses and physicians. Care providers are responsible for managing complex symptoms (e.g., refractory pain and multimorbidity), conducting advance care planning, and supporting patients and families during bereavement. Care consultants advise families on ethical issues, transitions, and advance care planning. Educators provide bedside teaching and disseminate palliative knowledge. Researchers contribute to palliative care research. Advocates contribute to raising awareness for palliative care.^[5]^

Quality of life has become an essential measure of well-being, particularly among palliative care professionals, whose clinical practice involves continuous exposure to suffering, serious illness, and end-of-life care, which may be associated with high levels of stress, burnout, and emotional burden, as well as reduced job satisfaction.^[6–8]^ Unlike isolated measures such as burnout or job satisfaction, QOL reflects a broader multidimensional construct encompassing physical, psychological, social, and environmental well-being. Poor QOL among healthcare workers has been associated with reduced work performance, impaired clinical decision-making, lower job satisfaction, absenteeism, and increased staff turnover, all of which may ultimately compromise the quality and continuity of patient care.^[6,9,10]^ Therefore, assessing QOL among healthcare professionals in the field of palliative care is not only vital for safeguarding their personal welfare but also important for supporting the delivery of high-quality care to patients.^[9]^

In Saudi Arabia, few studies have investigated the well-being of palliative care professionals despite their critical role. Ghazwani^[11]^ assessed the prevalence and risk factors of burnout among 44 palliative care physicians and documented emotional exhaustion in 18.2% of participants, as well as depersonalization, detachment, and reduced personal accomplishment in 25% each. Recently, Alhatim and AlShehery^[12]^ identified challenges perceived by Saudi palliative care physicians, emphasizing barriers such as restricted services, ethical dilemmas, and the absence of telemedicine. However, these studies were limited by small sample sizes and a narrow focus on specific professional groups, with limited attention to the broader concept of QOL among all palliative care professionals. Moreover, they primarily investigated challenges and barriers rather than comprehensively assessing QOL. Consequently, there remains insufficient evidence regarding the overall QOL of palliative care professionals in Saudi Arabia and the factors associated with it.

Given these gaps, a comprehensive assessment of QOL among palliative care professionals is needed to better characterize factors associated with their well-being. Such an assessment can help identify areas requiring organizational and psychosocial support, offering valuable insights for targeted interventions to enhance the well-being of palliative care professionals. Ultimately, such efforts may support improved staff retention, better patient care, and the overall enhancement of palliative care services. Accordingly, the present study sought to evaluate QOL among palliative care professionals in Tabuk Health Cluster hospitals and King Salman North West Armed Forces Hospital in Tabuk, Saudi Arabia, as well as to investigate the factors associated with QOL.

## 2. Methods

### 2.1. Study design, setting, and duration

This cross-sectional study was conducted over 9 months, from January to September 2025, a period considered sufficient to facilitate adequate participant recruitment. The study was carried out at 2 major healthcare institutions in Tabuk City, Saudi Arabia: Tabuk Health Cluster Hospitals and King Salman Northwest Armed Forces Hospital. Tabuk Health Cluster Hospitals are government-operated facilities under the Ministry of Health, providing comprehensive secondary and tertiary care services to more than 886,000 people through 84 primary healthcare centers and 12 general and specialized hospitals, with a total clinical capacity of 1488 beds. In contrast, King Salman Northwest Armed Forces Hospital is a leading tertiary care institution affiliated with the Ministry of Defense. It is accredited by the Joint Commission International and has a capacity exceeding 1000 beds, offering a wide range of advanced interventional and non-interventional subspecialties. The hospital also serves as a teaching center for medical students from Tabuk Medical College and is accredited by the Saudi Commission for Health Specialties, providing more than 10 residency programs and 2 fellowship programs. Both institutions function as major referral centers with diverse clinical departments, including palliative care services, and manage a substantial patient load. Including these settings enhances the representativeness of the study population and the generalizability of the findings.

### 2.2. Eligibility criteria

The study included male and female healthcare professionals from different specialties who were actively involved in providing palliative care at Tabuk Health Cluster Hospitals and King Salman Northwest Armed Forces Hospital in Tabuk, Saudi Arabia. Professionals who did not directly practice palliative care or declined to participate were excluded.

### 2.3. Sample size and sampling technique

The Raosoft online sample size calculator (https://raosoftcalculator.net/) was used to calculate the required sample size. Based on a 5% margin of error, a 90% confidence level, a 50% expected response distribution, and an estimated population size of 797 palliative care professionals across the included hospitals, the minimum required sample size was 260 participants. The estimated population size included healthcare professionals from multiple disciplines involved in providing palliative care within Tabuk Health Cluster Hospitals and King Salman Northwest Armed Forces Hospital. A non-probability convenience sampling method was used to recruit participants. Recruitment was conducted through the dissemination of an online survey link via institutional communication channels and professional social media platforms to maximize participation and facilitate recruitment.

### 2.4. Data collection tool

The English version of the World Health Organization QOL assessment-BREF (WHOQOL-BREF) was employed. It is a widely validated abbreviated version of the World Health Organization quality of life assessment (WHOQOL)-100 instrument and consists of 26 items. Two items assess overall QOL and general health, while the other 24 items are divided into 4 domains: physical health (7 items), psychological health (6 items), social relationships (3 items), and environmental health (8 items). The WHOQOL-BREF has demonstrated good validity and reliability across different populations.^[13,14]^

### 2.5. Calculation of domain scores

Each item within a domain was rated on a 1 to 5 scale, where 1 represented the poorest health status and 5 indicated the best. Items 3, 4, and 26 were reverse-coded. The domain score was computed by averaging the item scores (total points divided by the number [N] of questions) and then multiplying the mean by 4. This score was subsequently transformed into a 0 to 100 scale by subtracting 4 and multiplying the result by 6.25 (i.e., 100/16). On this final scale, 0 corresponds to the lowest possible QOL, and 100 reflects the highest.^[15]^

### 2.6. Data collection procedure

The questionnaire was converted into an online survey through the Google Forms platform. The survey link was disseminated to the 797 palliative care professionals across the included institutions via social media applications, predominantly WhatsApp, which is commonly used among healthcare professionals in Saudi Arabia. A final sample of 277 participants was obtained. Participants included both male and female healthcare professionals with varying levels of education and professional experience. The electronic survey included the WHOQOL-BREF instrument as well as items assessing participants’ sociodemographic and professional characteristics, including age, education level, occupation, medical specialty, healthcare sector, and marital status (single, married, divorced, or widowed). Participants were also asked to report any existing medical conditions at the time of the survey, which were considered self-reported baseline health status. The complete data collection form, including the item-level details of the WHOQOL-BREF, has been added to the supplementary files (Supplementary Table 1, Supplemental Digital Content 1).

Participants were informed that completing the questionnaire would take approximately 10 minutes. The survey link remained active for 4 weeks, allowing participants to complete it at their convenience. Two reminder messages were sent via the same social media channels to encourage participation. No financial or material reimbursement was provided, as participation was entirely voluntary and required informed consent.

### 2.7. Study outcomes

Quality of life, including physical health, psychological well-being, social relationships, and environmental health.

### 2.8. Ethical approval

This study was approved by the Institutional Review Board of the Ministry of Health, Tabuk Region, Saudi Arabia (Protocol No: TU-077/025/297; Approval Letter Date: 27 February, 2025), with expedited approval. All study procedures were conducted according to the approved documents, and participants provided informed consent.

Additionally, approval was obtained from the Research Ethics Team at King Salman Armed Forces Hospital, Tabuk, Saudi Arabia (Ethics ID: KSAFH-RET-2025–654), covering the research protocol, data collection tool, and consent process. The study adhered to all institutional policies, and participants’ confidentiality and data protection requirements were strictly maintained.

### 2.9. Statistical analysis

The data were tabulated and analyzed using IBM SPSS Statistics for Windows, version 27 (IBM Corp., Armonk). Internal consistency was assessed for the WHOQOL-BREF domains using Cronbach’s alpha. Categorical variables were summarized as frequencies and percentages, while numerical variables were assessed for distribution using skewness and kurtosis values, along with histograms, boxplots, and Q-Q plots. Since they followed a normal distribution, they were expressed as mean and standard deviation. Comparisons of QOL transformed scores across participants’ characteristics were conducted using either the independent samples *t*-test or 1-way analysis of variance (ANOVA), depending on the N of categories in the independent variables. When ANOVA showed significant results, Bonferroni post hoc pairwise comparisons were carried out. Either Cohen’s or eta-squared effect sizes were reported for the significant comparisons to show the magnitude of the effect. Furthermore, Pearson correlation analyses were performed to examine the relationships between age and the transformed scores of the 4 QOL domains. Age is an important demographic factor that may be associated with changes in QOL scores and is collected in this study as a continuous variable. Results were displayed as *r* correlation coefficients, their 95% confidence interval (CI), and *P*-values. Multivariable linear regression analyses were carried out to identify factors significantly associated with domain scores. A *P*-value <.05 was considered statistically significant.

## 3. Results

### 3.1. Reliability

The results showed Cronbach’s alpha coefficients for the 4 domains: 0.713 (0.644 − 0.771) for physical health, 0.694 (0.619–0.749) for psychological health, 0.695 (0.605–0.765) for social relationships, and 0.780 (0.721–0.825) for environment. The overall score showed a Cronbach’s alpha coefficient of 0.904 (0.882–0.922). This indicates good internal consistency (alpha > 0.70) for most domains.

### 3.2. Participant selection

A total of 797 healthcare professionals were eligible across the 2 institutions. The survey was completed by 277 participants, who were included in the final analysis.

### 3.3. Participant characteristics

The mean age of participants was 33.8 ± 6.3 years. More than half held a bachelor’s degree, while 35.4% were postgraduates. The participants were mainly nurses (48.4%), followed by residents (16.6%). Regarding specialty, internal medicine (35.4%) and critical care (21.3%) were the most common. About 2-thirds were employed in the Tabuk health cluster (66.1%), with the remainder in the Northwest Armed Forces Hospital (33.9%). More than half (56.0%) were married, while 31.8% were single. About 27% of participants had 1 or more current illnesses, the most common being diabetes mellitus (10.7%), hypertension, and anxiety (5.3% each) (Table 1).

**Table 1:** Sociodemographic and work characteristics of the study participants (n = 277).

		n = 277	%
Age, yr Mean ± SD		33.8 ± 6.3	
Education level	Bachelor	157	56.7%
	Post graduate	98	35.4%
	Diploma	21	7.6%
	Secondary school	1	.4%
Job	Nurse	134	48.4%
	Resident	46	16.6%
	Specialist	44	15.9%
	Consultant	28	10.1%
	Patient care technician	25	9.0%
Specialty	Internal medicine	98	35.4%
	Critical care	59	21.3%
	Oncology	45	16.2%
	Anesthesia	33	11.9%
	Palliative medicine	20	7.2%
	Pain medicine	13	4.7%
	Others	9	3.2%
Health sector	Tabuk health cluster	183	66.1%
	Northwest Armed Forces Hospital	94	33.9%
Marital status	Married	155	56.0%
	Single	88	31.8%
	Divorced	27	9.7%
	Widowed	7	2.5%
Current illness	No	202	72.9%
	Yes	75	27.1%
Type of illness	Unknown	40	53.3%
	Diabetes mellitus	8	10.7%
	Hypertension	4	5.3%
	Anxiety	4	5.3%
	Systemic lupus erythematosus	2	2.7%
	Hypothyroidism	2	2.7%
	Back pain	2	2.7%
	Asthma	2	2.7%
	Others*	11	14.7%

n = number, SD = standard deviation, Others: cardiology, neurology, gastroenterology, rheumatology, hematology, and general practitioner; Others*: toothache, osteoarthritis, epilepsy, abdominal pain, deep vein thrombosis, obesity, migraine, flu, rheumatoid arthritis, smoking, and fatigue. Current illness refers to self-reported preexisting medical conditions at the time of data collection.

### 3.4. General QOL and general health

More than half rated their overall QOL as good to very good, whereas satisfaction with general health was comparatively lower (only 41.6%). Detailed item-level WHOQOL-BREF response distributions are presented in Supplementary Tables 2 and 3, Supplemental Digital Content 2.

### 3.5. Physical health domain

The mean overall score of physical health was 51.7 ± 14.2. All items in this domain showed low ratings for good QOL or high satisfaction. The lowest-scoring items were sleep satisfaction, work capacity, ability to perform daily activities, and energy for everyday life (Supplementary Table 2, Supplemental Digital Content 2).

### 3.6. Psychological domain

In the psychological domain, the mean overall score was 48.7 ± 17.0. The highest-scoring items were concentration ability and enjoyment of life and sense of meaning, whereas the lowest-scoring items were the frequent experience of negative feelings such as anxiety or depression and lower satisfaction with bodily appearance and self-image (Supplementary Table 2, Supplemental Digital Content 2).

### 3.7. Social relationships domain

The mean overall social score was 45.9 ± 21.8. The lowest-scoring item was satisfaction with sex life, followed by personal relationships (Supplementary Table 3, Supplemental Digital Content 3).

### 3.8. Environmental domain

The mean overall environment score was 48.6 ± 16.4. Satisfaction with transportation emerged as one of the highest-scoring aspects, followed by the ability to meet daily financial needs and satisfaction with living conditions. In contrast, the lowest-scoring items were the physical environment and opportunities for leisure activities (Supplementary Table 3, Supplemental Digital Content 3).

### 3.9. Comparison of QOL domain scores according to the participants’ characteristics

Overall, the physical health domain had the highest mean transformed score (51.7 ± 14.2), while the social domain had the lowest mean transformed score (45.9 ± 21.8). Comparisons of the domain scores according to the participants’ characteristics revealed significant differences (Table 2).

**Table 2:** Comparison of physical health, psychological, social, and environmental quality of life scores according to the participants’ characteristics.

		Physical	Psychological	Social	Environment score	Quality of life score	Satisfaction with health
		Mean ± SD	Mean ± SD	Mean ± SD	Mean ± SD	Mean ± SD	Mean ± SD
**Job**	Specialist (n = 44)	55.1 ± 12.6	48.5 ±.11.8	47.9 ± 16.7	47.0 ± 14.4	47.7 ± 22.1	56.3 ± 26.5
	Resident (n = 46)	54.1 ± 15.0	49.2 ± 17.4	47.8 ± 22.7	54.1 ± 16.8	54.9 ± 20.2	54.9 ± 29.6
	Consultant (n = 28)	52.7 ± 14.9	55.1 ± 18.2	48.5 ± 23.8	49.9 ± 18.0	57.1 ± 22.4	56.3 ± 25.1
	Nurse (n = 134)	50.8 ± 13.9	47.9 ± 17.9	44.1 ± 22.8	47.0 ± 16.1	45.2 ± 26.6	50.8 ± 28.3
	Patient care technician (n= 25)	45.0 ± 13.9	45.5 ± 17.0	45.3 ± 21.0	48.5 ± 17.6	50.0 ± 30.6	47.0 ± 32.5
**AVOVA** ***P*-value**		0.037* η2 = 0.037	0.271	0.724	0.130	0.068	0.551
**Specialty**	Anesthesia (n = 33)	45.0 ± 13.7	43.3 ± 21.8	36.9 ± 19.9	41.6 ± 20.0	44.7 ± 31.1	38.6 ± 28.0
	Critical care (n = 59)	51.2 ± 13.9	49.3 ± 17.3	48.3 ± 22.9	45.6 ± 15.8	48.3 ± 23.2	55.5 ± 27.5
	Internal medicine (n = 98)	53.0 ± 12.9	48.8 ± 13.9	44.3 ± 19.6	49.6 ± 13.5	51.5 ± 20.9	57.1 ± 26.9
	Oncology (n = 45)	51.9 ± 13.9	50.7 ± 17.7	48.5 ± 22.8	51.3 ± 17.0	47.2 ± 29.8	51.7 ± 25.8
	Pain medicine (n = 13)	51.1 ± 14.6	47.1 ± 21.7	39.7 ± 24.6	45.2 ± 17.7	34.6 ± 26.1	42.3 ± 29.6
	Palliative medicine (n = 20)	54.1 ± 19.5	49.0 ± 18.1	53.8 ± 24.7	56.6 ± 19.0	46.3 ± 27.2	47.5 ± 34.3
	Others (n = 9)	59.9 ± 13.1	55.6 ± 9.5	58.3 ± 6.1	58.3 ± 12.5	72.2 ± 8.3	63.9 ± 30.9
**AVOVA** ***P*-value**		0.062	0.379	0.027* η2 = 0.051	0.005* η2 = 0.066	0.025* η2 = 0.052	0.018* η2 = 0.055
**Health sector**	Northwest Armed Forces Hospital (n = 94)	47.6 ± 14.1	42.0 ± 15.7	40.0 ± 20.8	44.1 ± 14.0	35.9 ± 24.8	42.3 ± 27.4
	Tabuk Health Cluster (n = 183)	53.8 ± 13.8	52.2 ± 16.6	48.9 ± 21.7	50.9 ± 17.1	55.5 ± 22.8	57.8 ± 27.3
**Independent Samples *t*-test** ***P*-value**		<0.001* Cohen’s *d*= 0.453	<0.001* Cohen’s *d* = 0.625	0.001* Cohen’s *d* = 0.417	<0.001* Cohen’s *d* = 0.424	<0.001* Cohen’s *d* = 0.822	<0.001* Cohen’s *d* = 0.566
**Marital status**	Single (n = 88)	53.7 ± 14.0	50.3 ± 17.7	42.7 ± 22.9	51.2 ± 16.9	47.2 ± 28.2	52.0 ± 29.9
	Married (n = 155)	51.2 ± 14.7	47.9 ± 17.1	49.0 ± 21.4	47.6 ± 16.5	50.5 ± 23.4	52.7 ± 27.9
	Divorced (n = 27)	48.4 ± 11.9	48.9 ± 16.1	38.6 ± 18.2	46.8 ± 15.1	44.4 ± 26.3	53.7 ± 28.3
	Widowed (n = 7)	51.0 ± 10.7	45.8 ± 4.8	44.0 ± 20.2	46.9 ± 10.2	50.0 ± 20.4	50.0 ± 20.4
**AVOVA** ***P*-value**		0.346	0.728	0.041* η2 = .030	0.354	0.598	0.986
**Current illness**	No (n = 202)	53.3 ± 14.2	49.8 ± 17.0	47.5 ± 21.9	49.6 ± 16.9	49.8 ± 23.9	56.4 ± 28.4
	Yes (n = 75)	47.5 ± 13.3	45.9 ± 16.7	41.4 ± 20.9	46.1 ± 14.7	46.3 ± 28.4	42.0 ± 25.4
**Independent Samples *T*-test** ***P*-value**		0.002* Cohen’s *d* = 0.417	0.092	0.039* Cohen’s *d* = 0.281	0.122	0.355	<0.001* Cohen’s *d* = 0.536
**Total**		51.7 ± 14.2	48.7 ± 17.0	45.9 ± 21.8	48.6 ± 16.4	48.8 ± 25.2	52.5 ± 28.3

n = number, SD = standard deviation;*: Significant at *P*-value <.05; Bold numbers indicate pairwise significant differences revealed by the Bonferroni post hoc test; Each pairwise comparison’s *P*-value was compared to this adjusted alpha to the number of comparisons; η2: eta squared effect size, which was reported for significant results only.

Job type showed a significant, small difference in physical health scores (*P*-value = .037, η^2^ = 0.037). Patient care technicians had significantly lower mean scores (45.0 ± 13.9) than specialists (55.1 ± 12.6). No other differences were observed in the remaining 3 domain scores across job roles.

For specialties, significant small to moderate differences were found in the social relationships (*P*-value = .027, η^2^ = 0.051), environmental (*P*-value = .005, η^2^ = 0.066), overall QOL (*P*-value = .025, η^2^ = 0.052), and satisfaction with health (*P*-value = .018, η^2^ = 0.055) scores. Notably, palliative medicine had the highest social (53.8 ± 24.7) and environmental (56.6 ± 19.0) scores. In comparison, anesthesia showed significantly lower social (36.9 ± 19.9), environmental (41.6 ± 20.0), and satisfaction with health (38.6 ± 28.0) scores. Pain medicine showed the lowest overall QOL rating (34.6 ± 26.1).

Regarding health sectors, participants from the Tabuk Health Cluster consistently scored higher across all domains than those from the Northwest Armed Forces Hospital (all *P*-values < .001), with small to large effect sizes (Cohen’s *d* ranging from 0.424–0.822).

Marital status showed a small but significant difference in social QOL scores (*P*-value = .041, η^2^ = 0.030), with married participants reporting higher social domain scores than divorced participants. However, no significant differences were observed across marital groups in the physical health, psychological, or environmental domains.

Regarding current illness, participants without a current illness had significantly higher scores in physical health (*P*-value = .002, Cohen’s *d* = 0.417), the social domain (*P*-value = .039, Cohen’s *d* = 0.281), and overall satisfaction with health (*P*-value < .001, Cohen’s *d* = 0.536). No significant differences were observed in the psychological or environmental domains or in the overall QOL rating between the 2 groups.

### 3.10. Correlation of domain scores and age

Age was not significantly correlated with the transformed scores of the physical health, psychological, social, or environmental domains. However, statistically significant positive moderate to large correlations were found between all 4 QOL domains. Physical health was significantly correlated with psychological (*R* = 0.629, *P*-value < .001), social (*R* = 0.470, *P*-value < .001), and environmental scores (*R* = 0.575, *P*-value < .001). Similarly, psychological scores showed significant correlations with social (*R* = 0.533, *P*-value < .001) and environmental domains (*R* = 0.708, *P*-value < .001). Social domain scores also correlated with environmental QOL (*R* = 0.584, *P*-value < .001; Table 3).

**Table 3:** Pearson’s correlation matrix between age and physical, psychological, social, and environmental quality of life scores.

		Age, yr	Physical health score	Psychological score	Social relations score
**Physical health score**	*r*	0.113	:	0.629	0.470
	95% CI	−0.005–0.228	–	0.552–0.695	0.373–0.490
	*P*-value	.060	–	<.001*	<.001*
Psychological score	*r*	0.085	0.629	–	0.533
	95% CI	−0.033–0.201	0.552–0.695	–	0.443–0.612
	*P*-value	.158	<.001*	–	<.001*
Social relations score	*r*	0.094	0.470	0.533	–
	95% CI	−0.024–0.209	0.373–0.490	0.443–0.612	–
	*P*-value	.119	<.001*	<.001*	–
Environment score	*r*	0.027	0.575	0.708	0.584
	95% CI	−0.091–0.144	0.490–0.649	0.501–0.644	0.501–0.657
	*P*-value	.657	<.001*	<.001*	<.001*

* : Significant at *P*-value <.05.

CI = confidence interval, *r* = Pearson’s correlation coefficient.

### 3.11. Multivariable regression

Multivariable linear regression models were conducted to assess factors significantly associated with the domain scores. All relevant participant characteristics were entered as independent variables in the model (inclusive models), including age, educational level, marital status, job, specialty, and current illness.

For the physical domain, participants with a current illness had significantly lower physical QOL scores compared with those without illness (Adjusted B = −4.56, 95% CI: −8.38–−0.73, *P* = .020). In the social domain, being unmarried (single/divorced/widowed) was associated with significantly lower domain scores compared with married participants (Adjusted B = −7.34, 95% CI: −12.96–−1.72, *P* = .011). In the environmental domain, participants working in palliative-related specialties (oncology, palliative medicine, and pain medicine) demonstrated significantly higher scores compared with those in anesthesia/critical care specialties (Adjusted *B* = 7.14, 95% CI: 2.20–12.08, *P* = .005). Meanwhile, no significant associations were observed between the participants’ characteristics and psychological domain scores.

Overall, all regression models explained a relatively small proportion of variance in QOL outcomes, with adjusted *R^2^* values ranging from 0.005 for the psychological domain to 0.045 for the physical domain. The physical and environmental domain models were statistically significant overall (*F*-test *P* = .013 and *P* = .020, respectively), whereas the psychological and social domain models were not statistically significant from the null model (Table 4).

**Table 4:** Multivariable linear regression for the 4 domains of WHOQOL-BREF.

Characteristic	Physical		Psychological		Social		Environmental	
	Adjusted B (95% CI)	*P*-value*	Adjusted B (95% CI)	*P*-value*	Adjusted B (95% CI)	*P*-value*	Adjusted B (95% CI)	*P*-value*
Age yr	0.24 (−0.10–0.58)	.165	0.12 (−0.3 –0.54)	.568	0.03 (−0.50–0.56)	.911	0.15 (−0.25–0.54)	.459
Highest education								
* Secondary school/Diploma*	–		–		–		–	
* Bachelor*	−1.35 (−8.87–6.17)	.724	0.63 (−8.62–9.88)	.894	−6.39 (−18.115.32)	.283	2.33 (−6.40–11.06)	.599
* Postgraduate*	−1.84 (−10.34–6.66)	.671	1.70 (−8.75–12.16)	.749	−6.87 (−20.11−6.38)	.308	7.20 (−2.66–17.07)	.152
**Marital status**								
* Married*	–		–		–		–	
* Single/Divorced/Widowed*	2.72 (−0.89–6.33)	.139	3.53 (−0.91–7.97)	.119	−7.34 (−12.96 – −1.72)	.011*	3.08 (−1.11–7.26)	.149
**Job**								
*Consultant/Specialist*	–		–		–		–	
* Nurse*	−2.63 (−9.27–4.02)	.437	−2.50 (10.67–5.68)	.548	−2.56 (−12.91–7.79)	.627	3.38 (−4.33–11.10)	.389
* Patient care technician*	–7.79 (–16.71–1.13)	.087	−3.87 (−14.84–7.11)	.488	−3.85 (−17.75–10.04)	.586	5.77 (−4.58–16.13)	.274
* Resident*	.34 (−6.50 – 7.18)	.922	−2.26 (−10.68–6.16)	.597	3.85 (−6.81–14.51)	.477	7.84 (−0.10–15.78)	.053
**Speciality**								
* Anesthesia/Critical care*	–		–		–		–	
* Medical-General*	2.17 (−2.0 –6.41)	.314	0.94 (−4.28−6.15)	.724	−1.46 (−8.06–5.14)	.664	4.14 (−0.78–9.06)	.099
* Palliative-Relevant*	3.53 (−0.73 – 7.79)	.104	2.43 (−.81−7.67)	.362	4.00 (−2.63–10.63)	.236	7.14 (2.20–12.08)	.005
**Current illness**								
* No*	–		–		–		–	
* Yes*	−4.56 (−8.38–−0.73)	.020	−3.45 (−8.17–1.26)	.150	−5.31 (−11.28–0.65)	.081	−2.21 (−6.66–2.23)	.328

Physical domain: Observations = 277; AICc = 2255; Adjusted *R^2^* = 0.045; F test (*P*-value) = .013.

Psychological domain: Observations = 277; AICc = 2370; Adjusted *R^2^* = 0.005; *F* test (*P*-value) = .580.

Social domain: Observations = 277; AICc = 2501; Adjusted *R^2^* = 0.022; *F* test (*P*-value) = .099.

Environmental domain: Observations = 277; AICc = 2338; Adjusted *R^2^* = 0.040; *F* test (*P*-value) = .020.

* *Significant at *P*-value < .05*.

CI = confidence interval.

## 4. Discussion

This study evaluated the QOL among palliative care professionals in Tabuk City, Saudi Arabia, and explored the factors associated with it. Among the 277 participants, the overall QOL was moderate, with the highest mean score observed in the physical domain, while the lowest was in the social domain. The relatively better physical health scores may indicate that participants generally maintained adequate physical functioning despite the demands of their work. In contrast, the low social domain scores indicate substantial challenges related to interpersonal relationships and social support, which are critical components of well-being in emotionally demanding fields such as palliative care. The lower social domain score may reflect the emotional demands and workload associated with palliative care practice; however, these factors were not directly measured in the present study. These observations suggest a potential need for organizational interventions to strengthen social support and support overall QOL among palliative care professionals. Our findings are consistent with previous studies that highlight the psychological and social burden experienced by palliative care professionals. Previous Saudi studies have documented high levels of emotional exhaustion and burnout among palliative care physicians, although QOL was not directly assessed in those studies. Ghazwani^[11]^ reported high levels of emotional exhaustion, depersonalization, and reduced personal accomplishment. Alhatim and AlShehery^[12]^ also identified systemic challenges such as limited services, ethical dilemmas, and a lack of supportive infrastructure, which may be relevant to the social and psychological well-being of palliative care professionals. These contextual factors may help explain the social and psychological scores observed in the present study. International studies similarly reported various levels of burnout and secondary traumatic stress among palliative care providers in a variety of settings. Studies from India,^[16]^ Spain, Brazil,^[17]^ the United States,^[18]^ and the Netherlands^[19]^ have reported differing levels of burnout, compassion satisfaction, and secondary traumatic stress. These differences have been attributed to variations in healthcare systems, training opportunities, workload distribution, and organizational support structures.

The item-level analysis provided further insights into the participants’ perceptions of QOL. Although more than half of the participants rated their overall QOL as good to very good, a considerably lower proportion reported satisfaction with their health. This discrepancy may indicate the presence of underlying physical or psychological strain despite a generally positive perception of QOL. This interpretation is supported by findings within the physical health domain, where reduced energy, sleep dissatisfaction, and work limitations were commonly reported, indicating substantial fatigue. In the psychological domain, scores for negative emotional states and body image satisfaction were less favorable. Socially, limited satisfaction with personal relationships and social support was evident. Environmental concerns included safety, financial resources, and opportunities for leisure. These patterns suggest that multiple dimensions of daily functioning may be affected, although cautious interpretation is recommended given the cross-sectional design of the present study and the reliance on self-reported data.

Another key finding in this study was the significant correlations among the 4 QOL domains, which is consistent with the broader literature. Studies involving oncology and critical care nurses have similarly reported that improvements in physical well-being are often accompanied by gains in psychological resilience, social functioning, and workplace satisfaction.^[20,21]^ Research among palliative care clinicians has also highlighted that stressors affecting 1 domain, such as emotional exhaustion, can negatively influence others, including social relationships.^[22]^ These findings indicate that interventions aimed at supporting palliative care professionals should adopt a comprehensive multidimensional perspective that addresses all QOL domains.

The present study also revealed important variations in QOL among palliative care professionals across different sociodemographic and occupational characteristics. Job type was significantly associated with physical health, where patient care technicians scored lower than specialists, which may reflect differences in the physical demands of their roles. Similarly, there were observed differences between specialties, with palliative medicine professionals scoring highest and anesthesia lowest in social and environmental domains. This result suggests that workplace culture, team dynamics, and exposure to emotionally challenging tasks may differ across disciplines and may be associated with variations in well-being; however, these factors were not directly assessed in this study. Furthermore, having an illness showed a negative association with the physical and social domains, which further reinforces the association between health status and professional QOL. In addition, married participants had better social domain scores than their divorced counterparts, possibly reflecting the potential contribution of social support to mitigating work-related stress. Consistent with our results, a Saudi study on nurses’ QOL during COVID-19 reported that married nurses had higher QOL scores than their single counterparts.^[23]^

The most consistent differences in this study were seen between health sectors. Professionals from the Tabuk Health Cluster reported significantly higher scores across all domains compared to those at the Northwest Armed Forces Hospital. This finding suggests that organizational structures, available resources, and institutional culture may be associated with the well-being of healthcare providers. However, because these organizational characteristics were not directly measured, their potential contribution should be interpreted cautiously.

In this study, participants’ age showed no significant correlation with any QOL domain. This suggests that age alone may not be a determining factor in professionals’ well-being and highlights the potential importance of broader organizational, social, and psychological factors. This finding is supported by the results of a previous Saudi study, which found no significant association between age and emotional exhaustion, depersonalization, or detachment.^[11]^ Similarly, research from India reported no association between age and the development of burnout or secondary traumatic stress.^[16]^ In contrast, Kamal et al ^[18]^ reported a higher prevalence of burnout among younger palliative care clinicians. Maqsood et al ^[23]^ also observed that older nurses had higher QOL scores, which they attributed to their greater professional experience and more developed coping strategies.

In the current study, after adjusting for potential confounders, the multivariable analysis revealed that only a limited N of factors were independently associated with WHOQOL-BREF domains. Current illness remained significantly associated with lower physical health scores, indicating that healthcare professionals with existing medical conditions reported lower physical QOL even after adjustment for sociodemographic and occupational factors. In contrast, most demographic variables, including age and educational level, were not independently associated with any QOL domain, suggesting that their observed relationships in unadjusted analyses may be explained by other overlapping factors. Marital status showed a domain-specific association, where being single, divorced, or widowed was independently associated with lower social relationship scores. This finding highlights the potential role of personal social support structures in shaping perceived social well-being, although a causal relationship cannot be established. Similarly, specialty was associated with environmental QOL, with professionals in palliative-related specialties reporting higher environmental scores compared with anesthesia and critical care. Critical care and anesthesia settings are typically characterized by higher patient acuity, time-sensitive decision-making, and increased workload intensity, which may contribute to differences in perceptions of the working environment. In contrast, palliative care settings often involve more structured care delivery and multidisciplinary collaboration, which may be associated with more favorable perceptions of environmental conditions. However, these interpretations remain exploratory, as specific workplace stressors and organizational characteristics were not directly measured in this study. The relatively low adjusted *R^2^* values across all domains indicate that a substantial proportion of variability in QOL remains unexplained. This suggests that additional unmeasured factors, such as workload intensity, burnout, team support, emotional demands, and organizational climate, may also be relevant contributors to QOL but were not captured in the present model.

Institutional and occupational variables showed more consistent associations than demographic factors, although the overall explanatory power of the models was limited. The relatively low adjusted *R^2^* values across all domains indicate that a substantial proportion of variability in QOL remains unexplained. This finding highlights the potential importance of additional unmeasured psychosocial and occupational factors, such as workload intensity, burnout, social support, and organizational climate. Future longitudinal studies incorporating these variables may provide further insight into their temporal relationships with QOL.

Our findings have practical implications for healthcare institutions and policymakers. Social domain scores were consistently lower. This finding suggests a potential need for interventions to strengthen interpersonal support and team cohesion within palliative care units. Potential strategies include peer support programs, regular debriefing after emotionally difficult cases, team communication training, mentorship for junior staff, and workload redistribution. At the institutional level, better staffing, adequate rest periods, and a supportive organizational culture may help support overall well-being. It may also be beneficial to integrate stress management and resilience-building into ongoing professional development. Importantly, targeted attention may be required for specific subgroups such as patient care technicians and professionals working in higher-risk specialties, who demonstrated relatively lower QOL scores. Future studies should also consider longitudinal assessment and incorporate psychosocial and occupational variables, including workload, burnout, social support, and organizational stressors, to better characterize factors associated with QOL.

## 5. Strengths

This study is strengthened by its comprehensive assessment of QOL among palliative care professionals across multiple specialties in 2 major hospitals, using the validated WHOQOL-BREF instrument. The sample size (n = 277) exceeds the minimum requirement, and participants were drawn from all major palliative care departments, supporting the coverage of diverse professional groups within the study settings.

## 6. Limitations

This study is limited by its cross-sectional design, which restricts the ability to draw causal inferences. The reliance on self-reported measures may also introduce response and recall biases, potentially influencing the accuracy of the reported QOL outcomes. Despite using a convenience sampling method, the included participants were drawn from all major palliative care departments across the 2 institutions, suggesting that the sample included a broad range of professionals within the participating institutions, although generalizability beyond these settings should be considered cautiously.

Another limitation of this study is the absence of data on participants’ sex, which prevented the examination of potential sex-based differences in QOL. This may be relevant, as previous evidence suggests that occupational stress, burnout, and well-being outcomes may vary according to sex. However, this variable was not available and therefore could not be included in the analysis. Also, no data were available on nonrespondents because of the online survey method, which prevented assessment of potential differences between participants and nonparticipants. Finally, the study did not account for potential confounding factors such as workload intensity, organizational support, or preexisting mental health conditions, all of which may have been associated with the observed QOL domains but were not assessed in the present study.

## 7. Conclusion

The findings indicate that palliative care professionals in Tabuk City experience a moderate overall QOL, with the social domain emerging as the most compromised. QOL varied significantly according to sociodemographic, occupational, and organizational characteristics, with certain subgroups reporting lower scores in specific domains. These findings suggest that organizational and psychosocial support may be relevant to the well-being of palliative care professionals.

Overall, the findings should be interpreted in the context of the cross-sectional design and should not be considered evidence of causal relationships.

## Author contributions

**Conceptualization:** Hanan Mohammad H. AlJammaz.

**Formal analysis:** Hanan Mohammad H. AlJammaz, Badaya Awadh M. Alshehri, Maram Ati Almohammadi, Fahad Abdullah Alqahtani, Ahmed Raja Albalawi, Rasha Ali Abdelrahman, Ahmed Abrahem Alhweti.

**Investigation:** Hanan Mohammad H. AlJammaz, Badaya Awadh M. Alshehri, Asma Ali Alharbi, Fahad Abdullah Alqahtani, Ahmed Raja Albalawi, Ahmed Abrahem Alhweti.

**Methodology:** Hanan Mohammad H. AlJammaz, Maram Ati Almohammadi, Asma Ali Alharbi, Ahmed Raja Albalawi.

**Data curation:** Badaya Awadh M. Alshehri, Asma Ali Alharbi, Fahad Abdullah Alqahtani, Ahmed Abrahem Alhweti, Rasha Ali Abdelrahman.

**Supervision:** Maram Ati Almohammadi, Asma Ali Alharbi.

**Writing – review & editing:** Maram Ati Almohammadi, Asma Ali Alharbi, Ahmed Raja Albalawi, Rasha Ali Abdelrahman.

**Writing – original draft:** Hanan Mohammad H. AlJammaz, Badaya Awadh M. Alshehri, Fahad Abdullah Alqahtani, Ahmed Abrahem Alhweti.






