# Supplementary material for: Assessment of quality of life among professional palliative care providers in Tabuk City, Saudi Arabia: A cross-sectional study
Source: Medicine (Baltimore). 2026 Oct 23;105(43):e51149. doi: 10.1097/MD.0000000000051149 (PMC13706271; doi:10.1097/MD.0000000000051149)
Supplement: Supplementary file 1 [file medi-105-e51149-s001.docx]

**Supplementary Table 1. Data Collection Form**

| **Section** | **Variable / Item** | **Response Options / Notes** |
| --- | --- | --- |
| **A. Sociodemographic & Work Characteristics** | Age | ___ years |
|  |  |  |
|  | Education Level | ☐ Diploma  ☐ Bachelor  ☐ Master  ☐ PhD  ☐ Others: _______ |
|  | Occupation / Job Type | ☐ Nurse  ☐ Physician  ☐ Specialist  ☐ Patient Care Technician  ☐ Other: _______ |
|  | Medical Specialty | ☐ Internal Medicine  ☐ Critical Care  ☐ Palliative Medicine  ☐ Anesthesia  ☐ Other: _______ |
|  | Healthcare Sector | ☐ Tabuk Health Cluster  ☐ King Salman Armed Forces Hospital |
|  | Marital Status | ☐ Single  ☐ Married  ☐ Divorced  ☐ Widowed |
|  | Presence of Current Illness | ☐ Yes (specify: _______)  ☐ No |
| **B. WHO Quality of Life Assessment – BREF (WHOQOL-BREF)** | **Instructions:** Please answer the following 26 items based on your experience over the past **two weeks**. Each item is rated on a **1–5 scale**, where 1 = Very poor / Very dissatisfied / Not at all, and 5 = Very good / Very satisfied / Completely. Items 3, 4, and 26 are **reverse-coded**. |  |
| **Domain 1: Physical Health (7 items)** | 1. How would you rate your physical health? | 1–5 |
|  | 2. Do you have enough energy for everyday life? | 1–5 |
|  | 3. Are you able to perform your daily living activities? | 1–5 |
|  | 4. How much do you need medical treatment to function in your daily life? | 1–5 |
|  | 5. How satisfied are you with your sleep? | 1–5 |
|  | 6. How satisfied are you with your ability to work? | 1–5 |
|  | 7. How satisfied are you with your capacity for physical activities? | 1–5 |
| **Domain 2: Psychological Health (6 items)** | 8. How much do you enjoy life? | 1–5 |
|  | 9. To what extent do you feel meaningful in life? | 1–5 |
|  | 10. How often do you feel negative emotions such as blue mood, despair, anxiety, depression? | 1–5 |
|  | 11. How much do you concentrate on what you do? | 1–5 |
|  | 12. How satisfied are you with your self-esteem? | 1–5 |
|  | 13. How satisfied are you with your ability to think, learn, and remember? | 1–5 |
| **Domain 3: Social Relationships (3 items)** | 14. How satisfied are you with your personal relationships? | 1–5 |
|  | 15. How satisfied are you with your sexual life? | 1–5 |
|  | 16. How satisfied are you with the support from friends? | 1–5 |
| **Domain 4: Environmental Health (8 items)** | 17. How satisfied are you with your physical environment (e.g., living conditions)? | 1–5 |
|  | 18. How satisfied are you with your financial resources? | 1–5 |
|  | 19. How satisfied are you with your freedom, physical safety, and security? | 1–5 |
|  | 20. How satisfied are you with health and social care accessibility and quality? | 1–5 |
|  | 21. How satisfied are you with your opportunities for acquiring new information and skills? | 1–5 |
|  | 22. How satisfied are you with your participation in and opportunities for recreation/leisure activities? | 1–5 |
|  | 23. How satisfied are you with your home environment? | 1–5 |
|  | 24. How satisfied are you with your transportation? | 1–5 |
| **General Items** | 25. How would you rate your overall quality of life? | 1–5 |
|  | 26. How satisfied are you with your health? | 1–5 |
