# Supplementary material for: Assessment of quality of life among professional palliative care providers in Tabuk City, Saudi Arabia: A cross-sectional study
Source: Medicine (Baltimore). 2026 Oct 23;105(43):e51149. doi: 10.1097/MD.0000000000051149 (PMC13706271; doi:10.1097/MD.0000000000051149)
Supplement: Supplementary file 2 [file medi-105-e51149-s002.docx]

**Supplementary Table 2. Responses of the participants to the general, physical health, and psychological aspects of quality of life**

|  | **1** | **2** | **3** | **4** | **5** |
| --- | --- | --- | --- | --- | --- |
|  | **n (%)** | **n (%)** | **n (%)** | **n (%)** | **n (%)** |
| How would you rate your quality of life? | 21 (7.6%) | 73 (26.4%) | 92 (33.2%) | 80 (28.8%) | 11 (4.0%) |
| How satisfied are you with your health? | 13 (4.7%) | 93 (33.3%) | 56 (20.2%) | 83 (30.0%) | 32 (11.6%) |
| **Physical Health Domain** | | | | | |
| To what extent do you feel that (physical) pain prevents you from doing what you need to do? **^a^** | 55 (19.8%) | 81 (29.2%) | 102 (36.8%) | 35 (12.6%) | 4 (1.4%) |
| How much medical treatment do you need any medical treatment to function in your daily life? **^a^** | 72 (26.0%) | 109 (39.4%) | 72 (26.0%) | 14 (5.1%) | 10 (3.6%) |
| Do you have enough energy for everyday life? | 20 (7.2%) | 73 (26.4%) | 111 (40.1%) | 63 (22.7%) | 10 (3.6%) |
| How well are you able to get around? | 15 (5.4%) | 60 (21.7%) | 105 (37.9%) | 82 (29.6%) | 15 (5.4%) |
| How satisfied are you with your sleep? | 45 (16.2%) | 107 (38.6%) | 76 (27.4%) | 43 (15.5%) | 6 (2.2%) |
| How satisfied are you with your ability to perform your daily living activities? | 22 (7.9%) | 77 (27.8%) | 106 (38.3%) | 62 (22.4%) | 10 (3.6%) |
| How satisfied are you with your work capacity? | 24 (8.7%) | 78 (28.2%) | 103 (37.2%) | 63 (22.7%) | 9 (3.2%) |
| **Psychological Domain** | | | | | |
| How much do you enjoy life? | 19 (6.9%) | 70 (25.3%) | 129 (46.6%) | 50 (18.1%) | 9 (3.2%) |
| To what extent do you feel your life is meaningful? | 28 (10.1%) | 69 (24.9%) | 110 (39.7%) | 44 (15.9%) | 26 (9.4%) |
| How well are you able to concentrate? | 19 (6.9%) | 54 (19.5%) | 139 (50.2%) | 50 (18.1%) | 15 (5.4%) |
| Are you able to accept your bodily appearance? | 39 (14.1%) | 60 (21.7%) | 101 (36.5%) | 49 (17.7%) | 28 (10.1%) |
| How satisfied are you with yourself? | 32 (11.6%) | 81 (29.2%) | 91 (32.9%) | 61 (22.0%) | 12 (4.3%) |
| How often do you have negative feelings such as a blue mood, despair, anxiety, or depression? **^a^** | 22 (7.9%) | 102 (36.8%) | 107 (38.6%) | 34 (12.3%) | 12 (4.3%) |

n: number; values are presented as frequency (percentage). Percentages are calculated per item and may not total exactly 100% due to rounding.1: Not at all/Never/Very dissatisfied/Very poor; 2: A little/Seldom/Dissatisfied/Poor; 3: Moderately/Quite often/Neither satisfied nor dissatisfied/Neither poor nor good; 4: Very much/Very often/Mostly/Satisfied/good; 5: An extreme amount/Always/Completely/Very satisfied/Very good; **^a^** reversed-coded items.
