# Supplementary material for: Assessment of quality of life among professional palliative care providers in Tabuk City, Saudi Arabia: A cross-sectional study
Source: Medicine (Baltimore). 2026 Oct 23;105(43):e51149. doi: 10.1097/MD.0000000000051149 (PMC13706271; doi:10.1097/MD.0000000000051149)
Supplement: Supplementary file 3 [file medi-105-e51149-s003.docx]

**Supplementary Table 3. Responses of the participants to the social and environmental quality of life items**

|  | **1** | **2** | **3** | **4** | **5** |
| --- | --- | --- | --- | --- | --- |
|  | **n (%)** | **n (%)** | **n (%)** | **n (%)** | **n (%)** |
| **Social Relations Domain** | | | | | |
| How satisfied are you with your personal relationships? | 23 (8.3%) | 78 (28.2%) | 103 (37.2%) | 51 (18.4%) | 22 (7.9%) |
| How satisfied are you with your sex life? | 57 (20.6%) | 64 (23.1%) | 90 (32.5%) | 48 (17.3%) | 18 (6.5%) |
| How satisfied are you with the support you get from your friends? | 23 (8.3%) | 75 (27.1%) | 100 (36.1%) | 51 (18.4%) | 28 (10.1%) |
| **Environment Domain** | | | | | |
| How safe do you feel in your daily life? | 23 (8.3%) | 61 (22.0%) | 119 (43.0%) | 48 (17.3%) | 26 (9.4%) |
| How healthy is your physical environment? | 32 (11.6%) | 78 (28.2%) | 121 (43.7%) | 34 (12.3%) | 12 (4.3%) |
| Do you have enough money to meet your needs? | 22 (7.9%) | 60 (21.7%) | 110 (39.7%) | 65 (23.5%) | 20 (7.2%) |
| How available to you is the information that you need in your day-to-day life? | 22 (7.9%) | 44 (15.9%) | 130 (46.9%) | 51 (18.4%) | 30 (10.8%) |
| To what extent do you have the opportunity for leisure activities? | 26 (9.4%) | 79 (28.5%) | 126 (45.5%) | 34 (12.3%) | 12 (4.3%) |
| How satisfied are you with the conditions of your living place? | 21 (7.6%) | 72 (26.0%) | 100 (36.1%) | 61 (22.0%) | 23 (8.3%) |
| How satisfied are you with your access to health services? | 29 (10.5%) | 58 (20.9%) | 110 (39.7%) | 58 (20.9%) | 22 (7.9%) |
| How satisfied are you with your transportation? | 28 (10.1%) | 51 (18.4%) | 94 (33.9%) | 62 (22.4%) | 42 (15.2%) |

n: number; values are presented as frequency (percentage). Percentages are calculated per item and may not total exactly 100% due to rounding.1: Not at all/Never/Very dissatisfied/Very poor 1: Not at all/Never/Very dissatisfied; 2: A little/Seldom/Dissatisfied; 3: Moderately/Quite often/Neither satisfied nor dissatisfied; 4: Very much/Very often/Mostly/Satisfied; 5: An extreme amount/Always/Completely/Very satisfied
